# Equivolumetric Protocol Generates Library Sizes Proportional to Total Microbial Load in 16S Amplicon Sequencing

**DOI:** 10.3389/fmicb.2021.638231

**Published:** 2021-02-26

**Authors:** Giuliano Netto Flores Cruz, Ana Paula Christoff, Luiz Felipe Valter de Oliveira

**Affiliations:** BiomeHub, Florianopolis, Brazil

**Keywords:** bacteria, absolute abundances, microbiome, colony-forming units, 16S rRNA, amplicon sequencing, Illumina

## Abstract

High-throughput sequencing of 16S rRNA amplicon has been extensively employed to perform microbiome characterization worldwide. As a culture-independent methodology, it has allowed high-level profiling of sample bacterial composition directly from samples. However, most studies are limited to information regarding relative bacterial abundances (sample proportions), ignoring scenarios in which sample microbe biomass can vary widely. Here, we use an equivolumetric protocol for 16S rRNA amplicon library preparation capable of generating Illumina sequencing data responsive to input DNA, recovering proportionality between observed read counts and absolute bacterial abundances within each sample. Under specified conditions, we show that the estimation of colony-forming units (CFU), the most common unit of bacterial abundance in classical microbiology, is challenged mostly by resolution and taxon-to-taxon variation. We propose Bayesian cumulative probability models to address such issues. Our results indicate that predictive errors vary consistently below one order of magnitude for total microbial load and abundance of observed bacteria. We also demonstrate our approach has the potential to generalize to previously unseen bacteria, but predictive performance is hampered by specific taxa of uncommon profile. Finally, it remains clear that high-throughput sequencing data are not inherently restricted to sample proportions only, and such technologies bear the potential to meet the working scales of traditional microbiology.

## Introduction

The application of high-throughput sequencing (HTS) methodologies allows large-scale identification of microorganisms, revealing colonization and dispersion patterns throughout studied sites such as hospitals, indoor or outdoor natural environments (Lax et al., 2017; Lloyd-Price et al., 2017; Thompson et al., 2017; Christoff et al., 2019; Gasparrini et al., 2019; Ribeiro et al., 2019). Despite various detailed microbiome characterization studies, most efforts address solely relative bacterial abundances within each sample, i.e., do not account for major variations of total microbial load (Vandeputte et al., 2017; Morton et al., 2019; Zemb et al., 2020).

Recent studies claim that the total number of reads in HTS-derived samples (library size) is an arbitrary sum, without biological relevance, yielding microbiome data as necessarily compositional in nature (Gloor et al., 2017; Jiang et al., 2019; Morton et al., 2019). Nonetheless, a previous study has demonstrated that library sizes need not be arbitrary, potentially holding significant correlations with input bacterial cell counts (Minich et al., 2018b).

The possibility of estimating absolute microbial abundance from HTS data has major impacts for research, government agencies, and industry, allowing researchers and policy makers to address microbiological issues in common scales, such as colony-forming units (CFU), without giving up the advantages of high-throughput technology. Further, relative information alone limits decision-making in scenarios in which sample microbe biomass is known to vary widely (Kembel et al., 2012; Oberauner et al., 2012; Mora et al., 2016). Bacterial percentages within a sample are hardly informative in terms of surface contamination levels or even risk of microbial environmental dispersion.

Notice that, here, we refer to “relative abundance” as taxa (potentially scaled) proportions or percentages within each sample, whereas the absolute abundance refers to the corresponding total CFU—although that ultimately refers to bacterial concentration, i.e., absolute abundance per sample. For instance, a sample with 50% of *S. aureus* and 10^2^ CFU is markedly different from another sample with the same percentage of *S. aureus* but 10^5^ CFU. We consider that these two samples differ in absolute abundance of *S. aureus*. We will keep this interpretation of the term “absolute abundance” throughout this manuscript. We further address potential terminology issues in the methods section “*A Note on Terminology.”*

The aim of this study is to demonstrate a library preparation strategy and statistical analysis approach that, together, are capable of estimating sample CFU using Illumina short-read sequencing data only—for both total microbial load and taxon-specific sample abundances. The approach herein described was primarily designed for the analysis of samples with varying total biomass (throughout this manuscript we refer to “biomass” as the sample bacterial biomass). Potentially, our method could be applied to hospital microbiome surveillance as well as other similar sampling sites with varying bacterial load and adapted to broader applications such as clinical evaluations and food safety management.

## Materials and Methods

### Samples

A synthetic DNA fragment with a naturally non-occurring sequence was designed with the 16S rRNA V3/V4 primers sequences flanking their extremities. This fragment with 544 bp was synthesized as gBlocks Gene Fragments from IDT (IA, United States). This DNA was eluted to a final concentration of 10 ng/μL in TE buffer following the manufacturer instructions. Then it was serially diluted from 0.56 to 0.00000056 ng/μL by a 10X factor dilution. This serial dilution is equivalent to a range of 954,000,000–954 copies of the synthetic DNA. Samples were processed in experimental triplicates.

Reference bacterial isolates were acquired from ATCC (American Type Culture Collection, VA, United States) ATCC 19111 *Listeria monocytogenes*, ATCC 14028 *Salmonella enterica*, ATCC 10876 *Bacillus cereus*, ATCC 12228 *Staphylococcus epidermidis*, ATCC 29212 *Enterococcus faecalis*, ATCC 8739 *Escherichia coli*, ATCC 25923 *Staphylococcus aureus*. These bacterial isolates were individually grown overnight at 35°C in Brain Heart Infusion media and then adjusted to an optical density (OD_600_) of 0.5, corresponding to 10^8^ CFU, to be further diluted with a 10X factor for more seven consecutive dilutions. The two more diluted concentrations for each bacterium had 100 μL plated in PCA (Plate Count Agar) and incubated overnight at 35°C to check for the CFU (Colony Forming Units) concentrations used in the assay described below. The dilutions corresponding to 2, 20, 200, 2,000, 20,000, and 200,000 CFU for each above bacterium were pipetted in a region of approximately 9 cm^2^ of a sterile plastic petri dish (90 × 15 mm) (Kasvi, Brazil), without media, and left to dry in a biological safety cabinet. Then the pooled bacterial cells were collected from the dry plate surface using a sterile hydraflock swab (Puritan, ME, United States) moistened with sterile physiological solution, for at least 1 min of swabbing. After sample collection the swab was broken down into a microtube containing 800 μL of stabilization solution–ZSample (BiomeHub, SC, BR). Collected samples were stored in room temperature for at least 24 h then after a vigorous vortexing, the swab was removed from the collection tube and the stabilization solution containing bacterial cells/DNA was used. The DNA from the above collected bacterial pools was extracted from the stabilization solution using a thermal lysis protocol (95°C for 10 min) followed by 1:1 AMPure XP magnetic beads purification (Beckman Coulter, CA, United States), two 200 μL ethanol 80% washes and ultrapure water elution in 40 μL. Samples were processed with fifteen replicates for each bacterial CFU dilution. Additionally, three alternative DNA extraction kits were used: QIAamp DNA Mini and Blood Mini (QIAGEN, Germany), lot: 154018620, used with protocol: DNA Purification from Blood or Body Fluids; DNAeasy Power Soil (QIAGEN, Germany), lot: 163024722 and DNAeasy Power Soil PRO (QIAGEN, Germany), lot: 160048809, both using 500 μL of stabilization solution as input and following the protocol as manufacturer instructions.

### Library Preparation and Sequencing

The 16S rRNA amplicon sequencing libraries were prepared using the V3/V4 primers (341F CCTACGGGRSGCAGCAG and 806R GGACTACHVGGGTWTCTAAT) (Wang and Qian, 2009; Caporaso et al., 2012) in a two-step PCR protocol. The first PCR was performed with V3/V4 universal primers containing a partial Illumina adaptor, based on TruSeq structure adapter (Illumina, United States) that allows a second PCR with the indexing sequences similar to procedures described previously (Caporaso et al., 2011). Here, we add unique dual-indexes per sample in the second PCR, also performing index switches between runs to avoid cross contaminations. Two microliters of individual sample DNA were used as input in the first PCR reaction. The PCR reactions were carried out using Platinum Taq (Invitrogen, United States) with the conditions: 95°C for 5 min, 25 cycles of 95°C for 45 s, 55°C for 30 s, and 72°C for 45 s and a final extension of 72°C for 2 min for PCR 1. For PCR 2, two microliters of the first PCR were used and the amplification conditions were 95°C for 5 min, 10 cycles of 95°C for 45.s, 66°C for 30.s, and 72°C for 45.s with a final extension of 72°C for 2 min. All PCR reactions were performed in triplicates. The second PCR reactions were cleaned up using AMPureXP beads (Beckman Coulter, United States) and an equivalent volume of each sample was added in the sequencing library pool. At each batch of PCR, a negative reaction control was included (CNR). The final DNA concentration of the library pool was estimated with Quant-iT Picogreen dsDNA assays (Invitrogen, United States), and then diluted for accurate qPCR quantification using KAPA Library Quantification Kit for Illumina platforms (KAPA Biosystems, MA). The sequencing pool was adjusted to a final concentration of 11.5 pM (for V2 kits) or 18 pM (for V3 kits) and sequenced in a MiSeq system (Illumina, United States), using the standard Illumina primers provided by the manufacturer kit. Single-end 300 cycle runs were performed using V2×300, V2×300 Micro, V2×500 or V3×600 sequencing kits (Illumina, United States) with sample coverages specified in Supplementary Table 1.

### Bioinformatics Analysis and Taxonomic Assignment

The sequenced reads obtained were processed using a bioinformatics pipeline described below. First, Illumina reads have the amplicon forward primer checked, it should be present at the beginning of the read, and only one mismatch is allowed in the primer sequence. The whole read sequence is discarded if this criterion is not met. The primers are then trimmed, and the reads accumulated error evaluated. Read quality filter (*E*) is performed converting each nucleotide Q score in error probability (e_*i*_), that is summed and divided by read length (L). The read is discarded if its accumulated error is above 0.35%.

(3) $$E=\frac{1}{L}\,\sum_{i=1}^{n}e_{i}E=\frac{1}{L}\,\sum_{i=1}^{n}e_{i}$$

(4) $$e_{i}=10^{-Q\,i/10}e_{i}=10^{-Q\,i/10}$$

Reads are then analyzed with the Deblur package v.1.1.0 (Amir et al., 2017) to remove possible erroneous reads and identical sequences are grouped into oligotypes (clusters with 100% identity). The sequence clustering with 100% identity provides a higher resolution for the amplicon sequencing variants (ASVs), also called sub-OTUs (sOTUs) (Knight et al., 2018)–herein denoted as oligotypes. Next, VSEARCH 2.13.6 (Rognes et al., 2016) are used to remove chimeric amplicons. We implemented an additional filter to remove oligotypes below the frequency cutoff of 0.2% in the final sample counts.

We also implemented a negative control filter for low biomass samples. If any oligotypes are recovered in the negative control results, they are checked against the samples and automatically removed from the results only if their abundance (in number of reads) are no greater than two times their respective counts in the sample. The remaining oligotypes in the samples are used for taxonomic assignment with the BLAST tool (Altschul et al., 1990) against a reference genome database (encoderef16s_rev6_190325, BiomeHub, SC, Brazil). This database is constructed with complete and draft bacterial genomes, focused on clinically relevant bacteria, obtained from NCBI. It is composed of 11,750 sequences including 1,843 different bacterial taxonomies.

Taxonomies are assigned to each oligotype using a lowest common ancestor (LCA) algorithm. If more than one reference can be assigned to the same oligotype with equivalent similarity and coverage metrics (e.g., two distinct reference species mapped to oligotype “A” with 100% identity and 100% coverage), the taxonomic assignment algorithm leads the taxonomy to the lowest level of possible unambiguous resolution (genus, family, order, class, phylum, or kingdom), according to similarity thresholds previously established (Yarza et al., 2014).

### Multiple Sequencing Correction (Normalization)

The correction procedure is fully described in Supplementary Material 1. Briefly, let $K_{j,i\in Q}^{n\,o\,r\,m}$ denote the normalized counts for the taxonomy *j* sample *i* ∈ *Q*, where *Q* is the set of samples from the *q*^*th*^ sequencing run. Then the normalization is a simply rescaling of the raw counts.

(5) $$K_{j,i\in Q}^{n\,o\,r\,m}=\frac{K_{j,i\in Q}}{S_{j,i\in Q}}$$

The size factor is sequencing-specific and is calculated as follows:

(6) $$S_{j,i\in Q}=S_{q}=\frac{\bar{A_{p^{*},q}}}{\max_{q^{\prime }=1,2,,m}\left(\bar{A_{p^{*},q^{\prime }}}\right)}$$

where $\bar{A_{p^{*},q^{\prime }}}$ is the average number of reads per sample made available a priori in the sequencing pool of interest *p*^⋆^ within sequencing *q*′ (expected sample coverage). The lower the relative availability, the smaller the resulting factor and thus greater the normalized values relative to the raw counts. Once normalized, divergences across samples from different sequencing runs, but of similar bacterial abundances, are assumed to rise mostly from sequencing efficiency differences–yet of negligible order of magnitude.

This procedure is markedly different from procedures from software packages such as DESeq2 or transformations from compositional data analysis, mostly proposed within the context of differential abundance analysis (Love et al., 2014; Quinn et al., 2019). Here, we only shift up read counts when the corresponding expected sample coverage is not equal to the maximum parameter within a given data set. While variation in relative availability does impact raw counts, it is likely innocuous with respect to the observed and herein addressed proportionality between read counts and sample absolute abundances.

### Statistical Analysis

All statistical analyses were performed using R software environment version 3.6.2 (Team, 2019). We used the brms R package and Stan (v. 2.11.1 and v. 2.19.1, respectively) to perform all Bayesian analyses and the tidyverse package suite (v. 1.3.0) for data wrangling and visualization (Bürkner, 2017; Carpenter et al., 2017; Wickham et al., 2019). We also used the phyloseq R package (v. 1.30.0) to handle microbiome data (McMurdie and Holmes, 2013). Supplementary Table 2 lists all R packages used along with corresponding versions and references. The entire modeling strategy is further detailed in Supplementary Material 2 (total microbial load) and 3 (bacterial abundances). All models were fit within the Bayesian framework.

### CPM for Total Microbial Load Estimation

We used a cumulative probability model (CPM) with a logit link, also known as Proportional Odds (PO) model, to predict total microbial load based on HTS reads. Let *Y_i* denote the total microbial load (in CFU scale) from the *i*^*th*^ sample. Given our serially diluted samples, we only observe *K* = 5 abundance values such that *Y_i* takes values *c*_*k*_ ∈ {*c*_1_,*c*_2_,…,*c*_*K*_}={0.84×10^2^,0.84×10^3^,…,0.84×10^6^}. We then define the model:

(7) $$Y_{i}∼C\,a\,t\,e\,g\,o\,r\,i\,c\,a\,l\,\left({\text{p}}_{i}\right)\,{\text{p}}_{i}={\left(p_{i\,1},p_{i\,2},p_{i\,3},p_{i\,4},p_{i\,5}\right)}^{T}$$

Each parameter is calculated as:

(8) pi⁢k=Pr(Yi=ck)pi⁢k=Pr(Yi=ck)=Pr(Yi≤ck)-Pr(Yi≤ck-1) for 1<k<K=Pr(Yi≤ck)-Pr(Yi≤ck-1) for 1<k<K

(9) $$p_{i\,1}=\text{Pr}\left(Y_{i}\leq c_{1}\right)p_{i\,1}=\text{Pr}\left(Y_{i}\leq c_{1}\right)$$

(10) $${\text{p}}_{\text{iK}}=1-\text{Pr}\left({\text{Y}}_{\text{i}}\leq {\text{c}}_{K-1}\right){\text{p}}_{\text{iK}}=1-\text{Pr}\left({\text{Y}}_{\text{i}}\leq {\text{c}}_{K-1}\right)$$

Finally, we compute the cumulative probabilities using ordinal logistic regression:

(11) $$logit\left[\text{Pr}\left({\text{Y}}_{\text{i}}\leq {\text{c}}_{\text{k}}\right)\right]=\psi_{\text{ik}},\text{for}\text{k}=1,2,\ldots ,K-1logit\left[\text{Pr}\left({\text{Y}}_{\text{i}}\leq {\text{c}}_{\text{k}}\right)\right]=\psi_{\text{ik}},\text{for}\,\text{k}=1,2,\ldots ,K-1$$

(12) $$\psi_{i\,k}=\alpha_{k}-\beta \cdot x_{i}\psi_{i\,k}=\alpha_{k}-\beta \cdot x_{i}$$

where *x_i* denotes the library size (total number of reads) for the observation *i*.

This generative model for the observed abundances *Y_i* is a case of ordinal logistic regression (McCullagh, 1985; Harrell, 2015). We use a logit link over the linear predictor ϕ_*ik*_ to estimate cumulative probabilities, i.e., $logit\left[\text{Pr}\left(Q_{i}\leq c_{k}|X=x_{i}\right)\right]=\psi_{i\,k}\Rightarrow \text{Pr}\left(Y_{i}\leq c_{k}|X=x_{i}\right)=\frac{1}{1\,e^{-\psi_{i\,k}}}$. The estimated cumulative probabilities originate the categorical parameters, and the resulting distribution then generates the observed data.

The linear predictor ψ_*ik*_ has two unknown parameters, the intercepts α_*k*_ and the slope β. We have placed weakly informative priors on both, with no prior preference for any class *c_k*:

(13) $$\alpha_{k}∼𝒩\,\left(0,5\right)\;a\,n\,d\;\beta ∼𝒩\,\left(0,5\right)$$

The intercepts are often called cutpoints as they represent the intersections between observable categories on the cumulative logit scale (Agresti, 2010b). Notice we set the same prior for all *K-1* cutpoints. The negative-valued slope parameter seen in Eq. 12 arises naturally from the PO model derivation with latent continuous variable motivation. It also guarantees intuitive interpretations: positive values indicate a positive effect toward higher categories (McElreath, 2018a).

The ordinal model also allows going beyond conditional (cumulative) class probabilities to estimate conditional expectations, quantiles, and tail probabilities (Harrell, 2015). This is a major advantage of CPMs over other more commonly used methods such as linear and quantile regression^30^. We fitted the model using brms and Stan (Bürkner, 2017; Carpenter et al., 2017).

### Hierarchical CPM for Absolute Bacterial Abundances

We develop a cumulative logit random effects model to predict bacteria-specific abundances based on observed HTS reads, which is basically a multilevel version of the previous model (7) (Agresti, 2010a). Let *Y*_*ij*_ denote the absolute abundance (in Colony-forming units) for the observation *i*, taxon *j*. Given our serially diluted samples, we only observe *K* = 4 abundance values such that *Y*_*ij*_ takes values *c*_*k*_ ∈ {*c*_1_,*c*_2_,*c*_3_,*c*_4_}={2×10^2^,2×10^3^,2×10^4^,2×10^5^}. We then define the model:

(14) $${\text{Y}}_{\text{ij}}∼C\,a\,t\,e\,g\,o\,r\,i\,c\,a\,l\,\left({\text{p}}_{\text{ij}}\right)\,{\text{p}}_{\text{ij}}={\left({\text{p}}_{\mathrm{ij1}},{\text{p}}_{\mathrm{ij2}},\ldots ,{\text{p}}_{\text{ijK}}\right)}^{\text{T}}$$

Except for the taxon subscript *j*, the parameters are computed according to Eqs (8) through (10), and the ordinal regression becomes:

(15) $$\text{logit}\left[\text{Pr}\left({\text{Y}}_{\text{ij}}\leq {\text{c}}_{\text{k}}\right)\right]=\psi_{\text{ijk}}\;\text{for}\text{k}=1,2,3\text{logit}\left[\text{Pr}\left({\text{Y}}_{\text{ij}}\leq {\text{c}}_{\text{k}}\right)\right]=\psi_{\text{ijk}}\;\text{for}\,\text{k}=1,2,3$$

(16) ψi⁢j⁢k=-jk⁢j⋅xi⁢jψi⁢j⁢k=-jk⁢j⋅xi⁢j

where *x*_*ij*_ is the number of reads in observation *i* from bacteria *j*. Differently from the previous model, here we have only four classes (*K* = 4) and hence *K*−1=3 cutpoints. We allow both intercepts and slopes to vary across bacteria, such that:

(17) $$\left[\begin{array}{c} \alpha_{k\,j}\\ \beta_{j} \end{array}\right]∼{𝒩}_{2}\left(\left[\begin{array}{c} \alpha_{k}\\ \beta \end{array}\right],\right)$$

Notice the mean of this two-dimensional Gaussian distribution is the vector of population-level parameters ^(α_*k*_β)*T*^. Thus, the variance-covariance matrix governs how the group-level parameters vary around the population-level counterparts:

(18) Σ=[σαk00σβ]⁢ℛ⁢[σαk00σβ]=[σk2σ,kβσ,kβσβ2]Σ=[σαk00σβ]⁢ℛ⁢[σαk00σβ]=[σk2σ,kβσ,kβσβ2]

(19) $$ℛ=\left[\begin{array}{cc} 1 & \rho \\ \rho & 1 \end{array}\right]ℛ=\left[\begin{array}{cc} 1 & \rho \\ \rho & 1 \end{array}\right]$$

We set the prior distributions for each unknown parameter:

(20) $$\alpha_{k}∼𝒩\left(0,2.5\right)\;\text{and}\;∼𝒩\left(0,2.5\right)\alpha_{k}∼𝒩\left(0,2.5\right)\;\text{and}\;∼𝒩\left(0,2.5\right)$$

(21) $$\alpha_{\text{k}}∼ℰxp\left(1\right)\;\text{and}\;∼ℰxp\left(1\right)\alpha_{\text{k}}∼ℰxp\left(1\right)\;\text{and}\;∼ℰxp\left(1\right)$$

(22) ℛ∼L⁢K⁢J⁢c⁢o⁢r⁢r⁢(2)ℛ∼L⁢K⁢J⁢c⁢o⁢r⁢r⁢(2)

The LKJ prior on the correlation matrix *ℛ* (which describes the correlation between α_*k*_ and *upbeta*) drives skepticism regarding extreme values near –1 and *1* (McElreath, 2018b). Jointly, the behavior of the prior distributions slightly favors outer categories (*k* ∈ {1,*K*}) in order to improve distinguishability for cases in which there were overlapping average number of reads. The prior choice was driven by model comparison using approximate leave-one-out cross-validation as well as prior and posterior predictive checks (Vehtari et al., 2017; Gabry et al., 2019).

### A Note on Terminology

Throughout this manuscript, we have termed as “absolute” the bacterial abundances in each sample as measured by their corresponding CFU. As previous works often refer to observed counts and observed proportions as proxies to relative bacterial abundances, we aim to avoid future confusion by clearly specifying the definitions herein employed.

Despite heterogeneity in the microbiome literature (Love et al., 2014; Morton et al., 2019; Calgaro et al., 2020), we call relative abundances any measure that is proportional to bacterial percentages within a sample. Recent reports have argued that HTS datasets are limited to this type of information because library sizes are arbitrary (Gloor et al., 2017; Quinn et al., 2018) at least under traditional (equimolar) protocols. To explicitly diverge from this scenario, we refer to the total CFU of a given taxon within a given sample as the taxon’s corresponding absolute abundance in that sample–a sample-wise notion of absolute abundance that has appeared previously in the literature (Williamson et al., 2019).

However, it is important to note that the sample absolute abundances are still relative to the sampled units, representing a measurement of concentration (e.g., CFU/μL). In the case of a well-defined system (e.g., a cell suspension of defined volume), in principle only by knowing both the sample and the system *sizes* (e.g., sample and suspension volumes, respectively) that we could recover the absolute abundances in the system as a whole (e.g., total CFU of *S. aureus* in the entire cell suspension), a potentially pernicious extrapolation we did not attempt. This makes knowing the system absolute abundances largely prohibitive in practice as it is often not clear how to perform such size measurements in a high-throughput fashion.

As an alternative, compositional data analysis techniques have been applied to uncover characteristics of a community that are mathematically equivalent whether we consider the sample proportions or the system absolute abundances, showing promising results (Morton et al., 2019; Quinn et al., 2019). During sample collection, however, it is frequently challenging to even define what a system is. For instance, when sampling a patient bed within a hospital, such entire system is hardly definable. The whole bed? The specific parts touched by patients or staff? Anything but the mattress? Noteworthy, the sample absolute abundance still yields insights limited to the sampling sites and immediately surrounding environment, which is easily interpretable.

Although we acknowledge there is room for terminology improvement and standardization, here we merely aim to stress the difference between learning that a sample shows 50% of a given taxon (sample relative abundance) and learning that the same sample has 10^4^ CFU of that same taxon (sample absolute abundance). While the chosen terminology attempts to state the difference between taxa percentages and taxa CFU within a sample, it is clear that the terms “relative” and “absolute,” when it comes to taxa abundances in general–whether in the sample or in a system as a whole -, require careful interpretation.

## Results

### Equivolumetric Protocol for Amplicon Library Preparation

**FIGURE 1:**
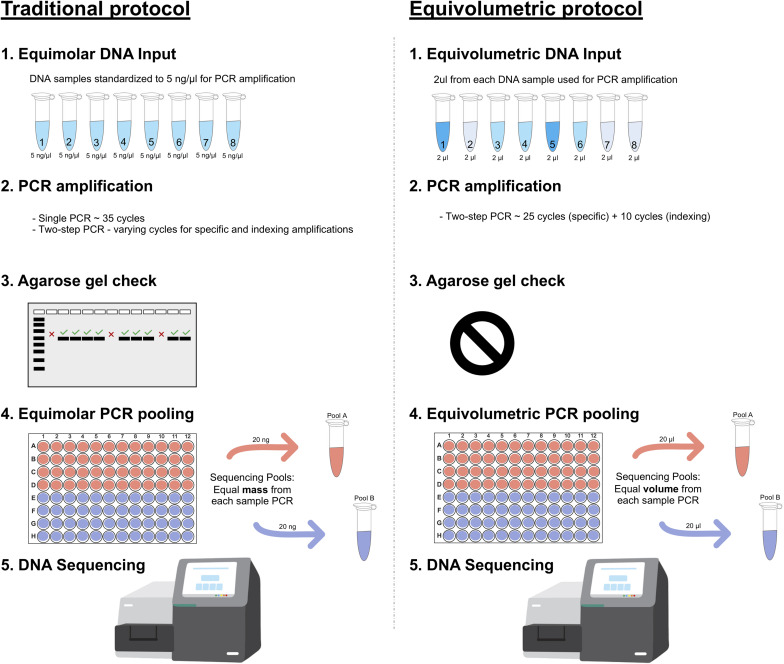
Amplicon library preparation methods for HTS sequencing. **Traditional protocol** is represented as the most common equimolar process. **(1)** Equimolar DNA inputs are prepared based on fluorimetric or spectrophotometric measures, all DNA samples are normalized to equivalent amounts (e.g., 5 ng/μL); **(2)** PCR amplifications are performed with single or two-step protocols with varying amplification cycles (most commonly 35 cycles); **(3)** Usually, PCR amplifications are then checked on agarose gel to confirm positive samples and discard negative ones; **(4)** PCR pooling for HTS sequencing is also performed in an equimolar manner through fluorometric quantification (e.g., pooling 20 ng from each sample). **Equivolumetric protocol** stands for equal volumes processed for each sample instead of equal concentration. In this protocol, samples retain their original differences in terms of concentrations of input DNA. **(1)** Equal volumes of each sample is used for PCR steps, regardless of its concentration (e.g., 2 μL); **(2)** Amplicon library preparation is carried out in a standardized, two-step PCR for 25 cycles using specific marker genes, then additional 10 cycles to add the sequencing adapter and indexes; **(3)** No agarose gel check is performed for these samples since we assume a wide variation in amplicon yield, related to the sample original DNA input; **(4)** PCR pooling for HTS sequencing is performed without specific sample normalizations. Equal volumes are used for each amplicon sample to assemble the HTS sequencing pool (e.g., pooling 20 μL from each sample).

In this study, we developed a customized laboratory protocol for 16S rRNA amplicon library preparation to recover absolute microbial abundances in each sample after Illumina short read sequencing (Figure 1). Briefly, we adapt traditional methods to handle unnormalized inputs of DNA and amplicon. While equimolar protocols standardize samples and PCR to fixed concentrations, we sample equal volumes of DNA or amplicon into each library preparation to keep major concentration differences intact. The PCR steps are also optimized for the same purpose, minimizing amplification cycles and stopping before most reactions plateau phase. Amplicon checking in agarose gel, capillary electrophoresis or other quantification method is not performed. In our protocol, final PCR pooling is also performed in an equivolumetric way, and DNA sequencing follows as in traditional methods using Illumina platforms.

### Input DNA and Absolute Bacterial Abundances

**FIGURE 2:**
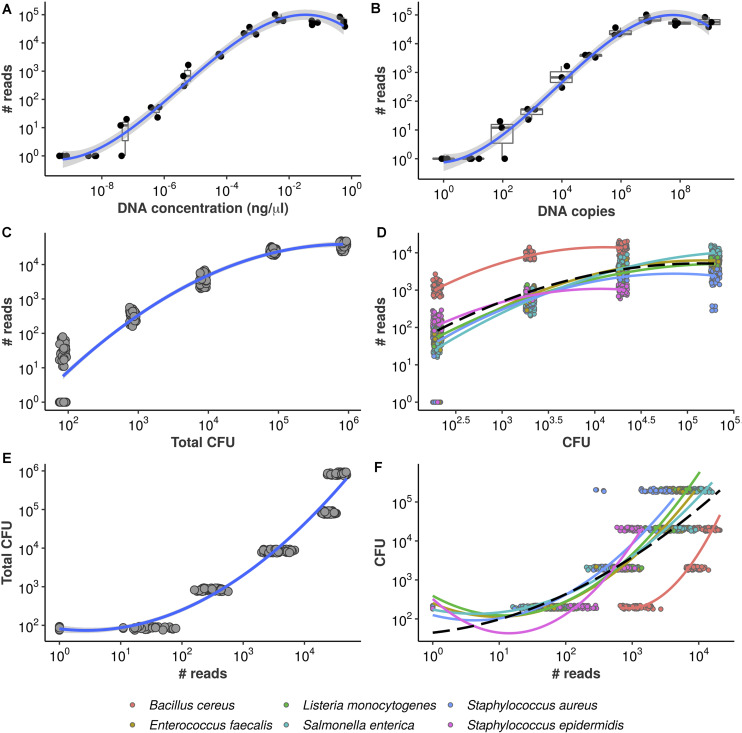
Equivolumetric protocol recovers proportionality between input DNA and HTS reads. Synthetic DNA fragment serially diluted from 0.56 to 0.00000056 ng/μL **(A)** or from 954,000,000 to 954 DNA copies **(B)** and its total number of reads obtained by HTS sequencing using the equivolumetric protocol. Total sample reads (library size) from sequencing of serially diluted samples of mock microbial community using the equivolumetric protocol demonstrates that the obtained read counts are proportional to total microbial load **(C)**. Similar relationship is observed between taxon-specific counts and abundances **(D)**. The estimation task of CFU based on HTS reads is illustrated for both total microbial load **(E)** and taxon-specific abundances **(F)**. Total microbial load ranged from 0.84*10^2^ to 0.84*10^6^ CFU, while taxon abundances ranged from 2*10^2^ to 2*10^5^ CFU. A pseudocount of 1 was added to the read counts to avoid *log*_10_(0).

To investigate whether our approach is capable of recovering absolute abundance information, we first assessed the relationship between generated reads and corresponding input DNA. We used a synthetic DNA molecule with known concentrations (Figure 2A) and sequenced replicated serial dilutions. A polynomial fit demonstrates the sigmoid trend, which indicates HTS-based quantification in absolute terms may still be bounded above by methodological constraints under our protocol (e.g., amplification plateau for highly concentrated samples). We also estimated the corresponding copy numbers and observed similar behavior (Figure 2B). Nonetheless, it is clear from this result that the total number of reads per sample (library size) increases with input DNA.

To further confirm the association between read counts and sample bacterial load, the sum of all bacteria CFU within a sample (also referred to as total microbial load), we sequenced serially diluted samples of known bacterial concentrations (in terms of CFU), mimicking a surface sample collection. Our results indicate that the equivolumetric protocol does recover proportionality between sequencing data and microbial load in terms of both library sizes (Figure 2C) and bacteria-specific counts (Figure 2D). We also demonstrate that this approach does not depend on DNA extraction method by testing four different extraction protocols and recovering sample abundances (Supplementary Figure 1).

### Using Data From Multiple Sequencing Runs

The replicates in Figures 2C,D come from four different sequencing runs, demonstrating the reproducibility of the method. As we keep total biomass differences, during data analysis we only correct for variations in the number of reads made available a priori for sequencing in each run (expected sample coverage, see section “Materials and Methods”). Yet, such variations hardly impact observed values across orders of magnitude on the *log*_*10*_ scale and are likely to have limited (if any) impact on the association between observed counts and corresponding bacterial abundance. Supplementary Material 1 describes such a correction in detail as well as an application using real hospital microbiome samples replicated through 14 sequencing runs.

### Proportionality Constraints and Limits of Quantification

From Figures 2A,B it can be noticed that the observed proportionality is limited within a specific range of DNA concentration or DNA copies–and, similarly, of bacterial abundance in CFU (Figures 2C,D). While lower values start failing to be detected by the sequencing–hampering quantification -, there are signs of saturation when DNA approaches 10^−2^ng/μL or 10^6^ copies as well as when bacterial abundance varies around 10^5^ and 10^6^ CFU.

Even though we do optimize the amplification steps for quantification, there is a threshold above which the amplification reaches a plateau phase—Supplementary Figure 2. This is the main reason behind the assumption that our protocol quantifies sample absolute abundances only within a limited range. By limiting the PCR cycles, we can detect major differences in input DNA during the exponential phase of the reactions, but not once the plateau is reached. Our PCR optimization, therefore, settles the upper bound of bacterial abundances that we are able to quantify. Above such point, all information generated is limited to the notion that the abundance of a given taxon (or the total microbial load) is greater than or equal to that upper limit of quantification.

### Modeling Absolute Abundance Using HTS Data

In the previous section, we presented data showing that HTS reads can respond monotonically to the increase of microbial load. By turning the axes around, we describe the present estimation task: given an observed library size (total sample reads), can total microbial load be predicted reliably? Figure 2E illustrates the problem.

Notice each value of microbial load varies only in orders of magnitude but corresponds to a relatively wide range of observed library sizes. Again, a polynomial trend is fitted, demonstrating the inadequacy of standard linear regression in this case. A similar behavior is observed if we analyze the read counts for each bacterium individually, despite significant taxon-to-taxon variation (Figure 2F). Such naive linear models ignore the monotonic, stepwise fashion in which total microbial load and absolute bacterial abundances vary conditionally on observed reads. Also, there are no prediction bounds: extrapolation toward higher library sizes yields continuously higher predicted values. This is likely unrealistic, given the plateaus observed in Figures 2A,B—and the inevitable PCR saturation as total sample biomass increases.

These characteristics led us to consider a cumulative probability model to robustly estimate total microbial load and absolute bacterial abundances using HTS data. In the next subsection, we briefly describe the fitted model for total microbial load–see section “Materials and Methods” for formal model specification. We then extend it onto a hierarchical structure that allows variation across bacteria in order to predict taxon-specific absolute abundances in each sample. Supplementary Materials 2, 3 describe both modeling strategies in further detail, including extensive prior and posterior predictive checks as well as assessment of modeling assumptions.

### Cumulative Probability Model Predicts Total Microbial Load

**FIGURE 3:**
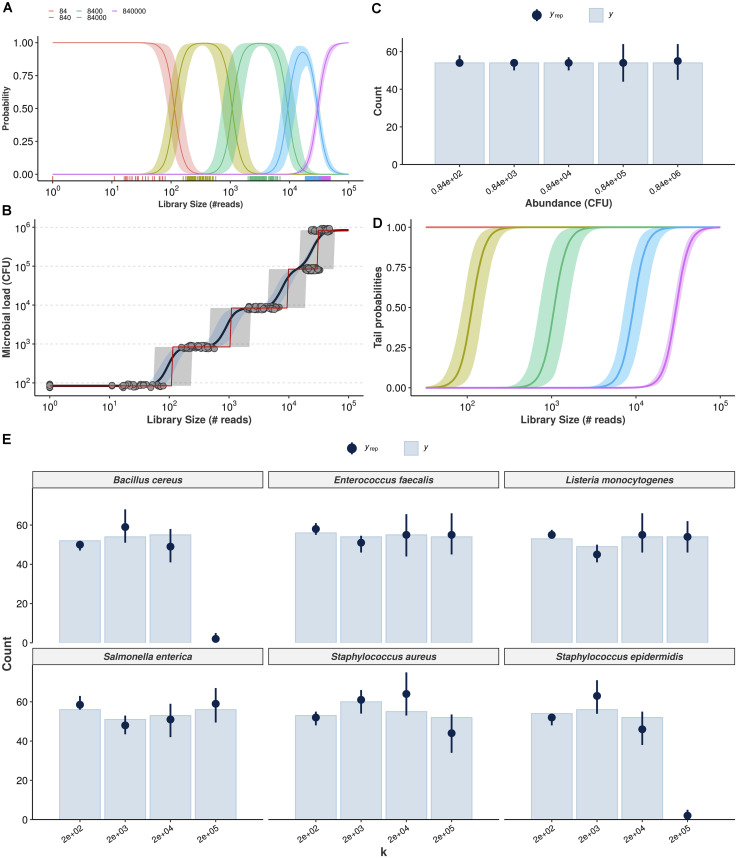
Cumulative probability models for the estimation of absolute bacterial abundances. Estimation of class probabilities for each observed value of total microbial load (in CFU), conditional on observed library size, is retrieved from the ordinal logistic regression framework **(A)**. Conditional expectations are then derived as weighted average of microbial load values and respective class probabilities (black line, 95% credible intervals in light blue) **(B)**. The class of highest probability (CHP, the most likely outcome given the observed reads) is also shown (red line, 95% predictive intervals in gray). Posterior predictive check shows the Bayesian model captures the overall structure of the observed data for total microbial load (*y*_*rep*_: posterior draws, *y*: observed data) **(C)**. Tail probabilities, herein defined as the probability of observing at least class ck, conditional of observed library size are an alternative for cases in which CHP- and expectation-based predictions are prohibitively uncertain **(D)**. Hierarchical CPM accounts for differences across bacteria and takes advantage of partial pooling to estimate taxon-specific abundances **(E)**. The resulting posterior predictive check indicates no major signs of misfit.

To predict the total microbial load in a sample, in terms of colony-forming units, we propose a Bayesian cumulative probability model (CPM) with logit link, also known as Proportional Odds (PO) model—a case of ordinal logistic regression. See section “Materials and Methods” for the entire model specification and Supplementary Material 2 for the detailed workflow. Figures 3A–D show the results.

Figure 3A shows the estimated class probabilities as a function of library size, and Figure 3B shows the implied expectations (black solid line, 95% credible intervals in blue) as well as the class of highest probability (CHP, red solid line). Predictive intervals for the CHP are also shown in light gray. Notice how the red line in Figure 3B follows closely the behavior of the class probabilities in Figure 3A. The predictions generated by the ordinal model are by construction monotonic. Also note that both expectation and CHP are bounded within the observed outcome range, overcoming extrapolation issues related to the previous naive model. In Figure 3C, posterior-predictive check indicates the overall structure of the observed data is well captured by the posterior draws of the model. Finally, Figure 3D shows model-implied tail probabilities, herein defined as the (conditional) probability of observing at least abundance *c_k*, i.e., *Pr*(*Y*_*i*_≥*c*_*k*_|*X* = *x*_*i*_) rather than *Pr*(*Y*_*i*_ > *c*_*k*_|*X* = *x*_*i*_). In the next subsection, we extend the previous model to handle taxonomic information in a hierarchical fashion so that one can make predictions of absolute abundance for each bacterium individually. We then validate both models using cross-validation and prediction on held-out samples (test sets) in the following subsection.

### Hierarchical CPM Predicts Absolute Bacterial Abundances

In order to handle taxonomic information, we formulate a similar model which incorporates taxon-specific effects in a hierarchical structure. The major difference is that the linear predictor term is parametrized with population-level parameters and group-level counterparts (for both intercept and slope), allowing predictions of many bacteria with a single model and taking advantage of partial pooling (Agresti, 2010a).

While we use seemingly weakly informative priors (see section “Materials and Methods” for full model specification), their joint behavior favors outer classes to improve distinguishability when dealing with classes of overlapping average number of reads. This is illustrated with prior-predictive check and assessment of CPM assumptions in Supplementary Material 3, which also shows detailed workflow and visualizations for each observed bacterium. Our prior choice resulted from model comparison with approximate leave-one-out cross-validation (Vehtari et al., 2017; Gabry et al., 2019). Figure 3E shows the corresponding posterior predictive check, suggesting the data is well captured by the model-implied data generation process for all bacteria. *B. cereus*, *S. aureus*, and *S. epidermidis* may represent challenging cases, although this can be an artifact from estimating varying effects with only six bacteria (stronger priors led to worse fit during model comparison).

### Model Validation

We validated both cumulative probability models for total microbial load and taxon-specific abundances using 10-fold cross-validation (CV) and prediction on held-out test sets comprising of 10% of the total number of observations. We assess performance both as classification and regression tasks–using CHP- and expectation-based predictions, respectively.

**FIGURE 4:**
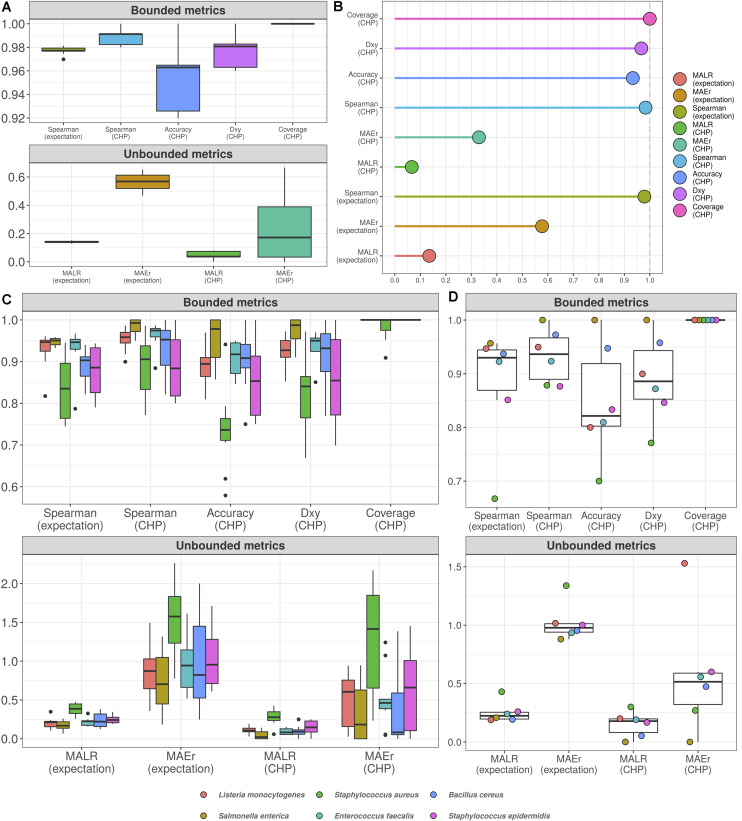
Cumulative probability models generate accurate predictions for total microbial load and taxon-specific absolute abundances. Performance measures from 10-fold cross-validation of total microbial load model indicate predictive errors are constrained far below one order of magnitude **(A)**. For visualization, bounded metrics vary between 0 and 1, while unbounded metrics vary in the positive real line. Similar results were observed in the held-out test set **(B)**. 10-fold cross validation for taxon-specific predictions using hierarchical CPM indicates predictive performance varies across bacteria, although still far below one order of magnitude **(C)**. Similar results were observed in the held-out test set **(D)**. Predictions based on class of highest probability are indicated with (CHP) in the *x*-axis, and expectation-based counterparts are indicated likewise. MALR: mean absolute log-ratio; MAEr: mean absolute error relative to true values; *D*_*xy*_: Somers’ Delta measure of ordinal association; Coverage: observed coverage of 95% predictive interval.

Figure 4A shows the 10-fold CV results for the total microbial load model. For visualization, we have split the assessed metrics into bounded between 0 and 1 and unbounded metrics. Bounded metrics based on CHP included the observed coverage of 95% predictive interval, Somers’ Delta (measure of ordinal association), classification accuracy, and Spearman’s rank correlation. The latter was also assessed for expectation-based predictions. In general, these metrics varied well above 0.9. Notably, the predictive intervals showed 100% coverage, which is likely overconfident. Nonetheless, most intervals spanned two abundance classes as in Figure 3B (see also Supplementary Materials 2, 3), suggesting errors occur mainly within one order of magnitude from the true values. Ordinal association, as measured by Somers’ Delta, was consistently greater than 0.95 for both CV and test set.

Unbounded metrics relied on modified versions of absolute errors, for both CHP- and expectation-based predictions. MALR and MAEr denote Mean Absolute Log-Ratio and Mean Absolute Error relative to true value, respectively, defined as follows.

(1) $$M\,A\,L\,R=\frac{1}{n}\,\sum_{i=1}^{n}\left|l\,o\,g_{10}\,\left({\hat{y}}_{i}\right)-l\,o\,g_{10}\,\left(y_{i}\right)\right|=\frac{1}{n}\,\sum_{i=1}^{n}\left|l\,o\,g_{10}\,\left(\frac{{\hat{y}}_{i}}{y_{i}}\right)\right|M\,A\,L\,R=\frac{1}{n}\,\sum_{i=1}^{n}\left|l\,o\,g_{10}\,\left({\hat{y}}_{i}\right)-l\,o\,g_{10}\,\left(y_{i}\right)\right|=\frac{1}{n}\,\sum_{i=1}^{n}\left|l\,o\,g_{10}\,\left(\frac{{\hat{y}}_{i}}{y_{i}}\right)\right|$$

(2) $$M\,A\,E\,r=\frac{1}{n}\,\sum_{i=1}^{n}\frac{\left|{\hat{y}}_{i}-y_{i}\right|}{y_{i}}M\,A\,E\,r=\frac{1}{n}\,\sum_{i=1}^{n}\frac{\left|{\hat{y}}_{i}-y_{i}\right|}{y_{i}}$$

MALR represents deviance in orders of magnitude, which varied during CV below 0.2 for both CHP and expectation, a result reproduced in the test set evaluation (Figure 4B). Perhaps more intuitive, MAEr represents absolute errors as proportions of true values and tended to be smaller for CHP-based predictions compared to expectations. During 10-fold CV or test-set validation, we did not observe MAEr values greater than 0.7.

Although not as common as mean absolute errors or mean squared errors, the metrics herein assessed do not penalize estimation in varying orders of magnitude, offering advantages in interpretation. A MALR value of 1 corresponds to a ratio between predicted and observed values of one order of magnitude in the *log*_*10*_ scale. A MAEr of 1 indicates prediction absolute error as large as the true value, which would still be largely insignificant given the logarithmic scale. Using both CHP- and expectation-based predictions, our results indicate that predictions for the total microbial load model were mostly kept within the observed orders of magnitude.

Figure 4C (10-fold CV) and Figure 4D (test-set) show the analogous measures for the hierarchical model with taxon-specific predictions. Median MAEr varied below 1 for both CHP and expectations during CV for most bacteria. In the test set, the highest value observed was close to 1.5 (*L. monocytogenes*). We observed most median accuracy values as high as 0.85 during CV and 0.8 for the test set, while ordinal association seems slightly higher in general. The model was least performant for predicting *S. aureus* abundance, as indicated by almost all metrics computed. Still, observed MALR varied consistently below 0.5 for all bacteria both in CV and test-set validation. Again, the results indicate our predictions are contained within respective orders of magnitude, suggesting that HTS reads can indeed be a valuable source of information regarding absolute bacterial abundances.

### Predicting Abundance of Previously Unseen Bacteria

As the hierarchical CPM enables prediction of previously unseen bacteria, we also performed leave-one-group-out CV to assess how our model could generalize in high-throughput settings, in which one may have dozens of taxa of interest. For each bacterium, we hold out its corresponding data points and train a separate model with the remaining data. We then perform predictions for the held-out taxon, treating it as “previously unseen”–not used during model fitting.

**FIGURE 5:**
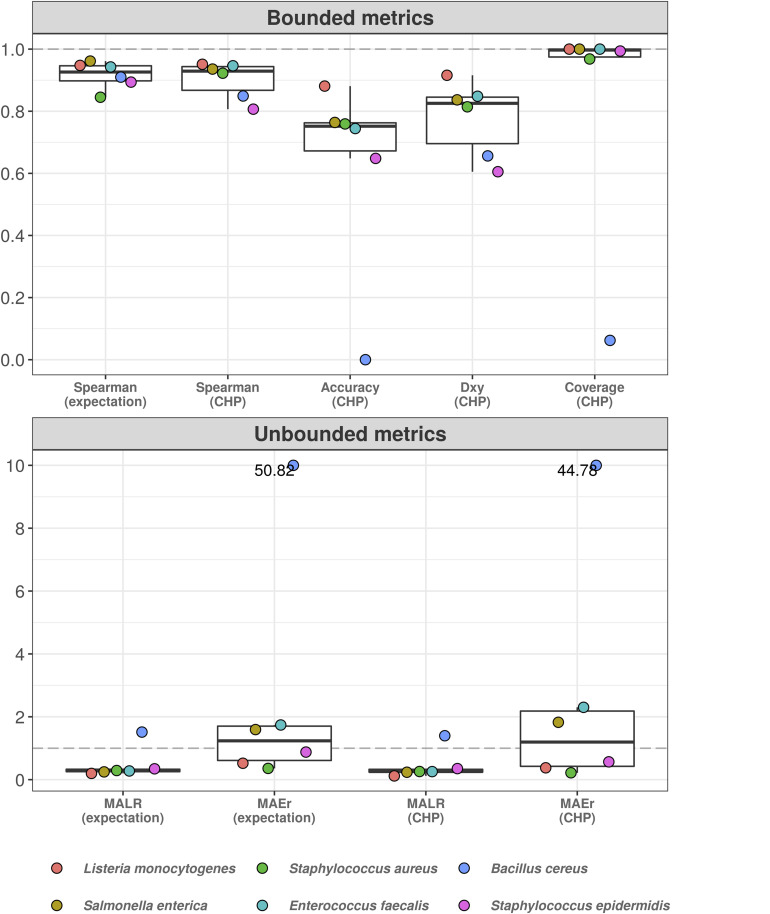
Hierarchical cumulative probability model predicts previously unseen bacteria with varying performance. Leave-one-group-out cross-validation was used to estimate predictive performance of hierarchical CPM for previously unseen bacteria. The predictive errors are constrained below one order of magnitude for most bacteria, except for *Bacillus cereus*–which reached errors of almost two orders of magnitude (lower panel). The dashed gray line indicates a value of 1, representing one order of magnitude in the context of MALR. The model fails to classify abundance values of *Bacillus cereus* (upper panel), although ordinal association (*D*_*xy*_) remains above 0.6. Most absolute errors represent no more than two times the observed abundances in a context of logarithmic differences–except for *B. cereus* and (slightly) *E. faecalis* using CHP. For visualization, we truncated the *y*-axis of the lower panel at the value of 10 and indicated higher values with numeric labels.

Figure 5 shows the results. Classification accuracy drops substantially, and the model completely fails to classify abundance values for *B. cereus*. Yet, for other bacteria, ordinal association and classification accuracy varied between 0.9 and 0.6. Treated as a classification task, the poor predictive performance is likely influenced by high uncertainty associated with the estimation of varying intercepts and slopes using data from only five bacteria at each iteration. This can also explain the high coverage values: predictive intervals were so wide that potentially spanned nearly all outcome space. On the other hand, the taxon associated with the worst out-of-sample performance (*B. cereus*) was also the one with the highest random effects in the original model, i.e., the greatest deviance from the overall, population-level effects (see Supplementary Material 3). While most bacteria showed MAEr between 0.5 and 3, *B. cereus* exceeded the value of 50 (absolute error as large as 50 times the true value). Nonetheless, MALR still varied below the threshold of 1 for all but *Bacillus cereus*, which almost reached a MALR of 2 (two orders of magnitude).

## Discussion

### Equivolumetric Protocol for 16S rRNA Sequencing

Here we have shown that assessment of absolute bacterial abundance using 16S amplicon short-read sequencing becomes possible upon protocol alteration within specified conditions, and the remaining challenges lie within the realm of resolution and taxon-to-taxon variation. While library sizes do depend on sequencing effort–a partially arbitrary sequencing setup -, equivolumetric protocols assure the maintenance of major input DNA variations, at least for certain ranges of absolute abundance.

The major difference in our equivolumetric protocol relies in the absence of DNA/amplicon normalizations, while equimolar protocols standardize samples and PCR to fixed concentrations (Caporaso et al., 2011; Gohl et al., 2016; Minich et al., 2018a; Chen et al., 2019; Ribeiro et al., 2019; Sielaff et al., 2019). Also, we use fewer PCR steps, potentially decreasing error rates and chimera formation, as previously reported (Gohl et al., 2016; Sze and Schloss, 2019). After library amplification, the agarose gel check or capillary electrophoresis can be useful for samples with sufficient biomass, though it is often the case that low biomass samples will show no results, hampering any useful interpretation (Minich et al., 2018b). For this reason, we do not check for amplicons presence or concentration after library preparation.

The main goal of our method is to quantitatively assess the microbiome of indoor environments, such as hospital surfaces, that are generally characterized by low biomass samples, despite wide variation (Kembel et al., 2012; Oberauner et al., 2012; Mora et al., 2016; Minich et al., 2018b). Still, this is only one scenario in which quantitative information regarding absolute abundance can be especially useful. Other studies have performed shotgun metagenomic sequencing, which could also be an informative way to characterize bacterial abundances. However, metagenomic sequencing of low-biomass samples is particularly challenging due to insufficient microbial DNA (Minich et al., 2018b). Importantly, metagenomics is very costly compared to amplicon sequencing, thus rendering implementation in large experimental settings even more difficult.

A key assumption for sequencing normalization across runs is that the expected sample coverages are not underestimated for the current quantification range so that the read counts are not censored due to low availability of reads or library saturation. While more sophisticated data transformations may be needed for other situations, when varying biomass by orders of magnitude such a step is largely simplified under our protocol. Even though we can still refer to the proposed procedure as normalization, it is markedly different from methods employed by software packages such as DESeq2 and EdgeR in the context of differential expression/abundance analysis (Robinson et al., 2010; Love et al., 2014). While we do log-transform the output reads, our normalization merely scales up the read counts from pools of samples with lower expected sample coverages, thereby allowing the use of data from different sequencing runs to make predictions. Importantly, we can annulate the effect of normalization by using data from a single run, given that all samples were sequenced with the same expected coverage, or similarly, by keeping the expected sample coverage constant across all sequencing runs. We do not assume, therefore, any dependency between such a procedure and the observed proportionality between reads and absolute abundances.

### Proportionality Between HTS Reads and Absolute Abundance

PCR amplification steps are well-known methodological constraints in 16S rRNA sequencing (Kebschull and Zador, 2015). However, a previous study already showed closed relationship between high-throughput sequenced reads and total bacterial cells (Minich et al., 2018b). Here we showed that while PCR saturation and sample sequencing coverage can still impose upper quantification limits, the proportionality of sequenced reads was consistently reproduced with respect to known DNA concentrations, DNA copies, and CFU among the samples. Lower limits of quantification can also be optimized further by testing a finer grid of concentrations in the lowest range, experimentation that goes beyond the scope of this study.

Quantification of absolute abundances using 16S rRNA gene amplicon sequence data has been object of study in the recent microbiome literature (Vandeputte et al., 2017; Morton et al., 2019; Zemb et al., 2020). One way to recover absolute bacterial abundance for each sample is through association of relative information from HTS technology with absolute information from other methods. This has been previously done using qPCR or flow cytometry as absolute abundance methodologies and HTS as provider of bacterial proportions (Vandeputte et al., 2017; Williamson et al., 2019; Zemb et al., 2020). Here, however, by fixing volumes rather than concentrations during library preparation, our strategy allows the detection of major variations in input DNA within a specified quantification range. Still, estimation of sample absolute abundances remains challenged by resolution and taxon-to-taxon variation: a smooth continuum of sample CFU values is hardly observable in practice, and the expected value of read counts given a value of CFU is notably variable across bacteria.

In the equivolumetric protocol, as we still keep the ability to detect bacterial proportions–despite known biases (McLaren et al., 2019)-, we maintain the information retrieved under equimolar protocols, even if a sample is outside the absolute quantification range. Additionally, if a sample shows too high biomass, the CPM will predict a high probability of having at least the upper limit of quantification in terms of total microbial load. Similarly, samples with very low biomass will yield a high probability of having at most the lower limit of detection. This flexibility to work with tail probabilities and to constrain estimation within the range of previously observed abundances is a key advantage of employing a CPM. Using our proposed approach, hence, total microbial load potentially gains importance to be applied for surveillance of indoor environments and similar sampling sites as a measure of total contamination. The main justification for our proposal is the fact that samples from indoor environments vary widely in terms of total biomass, generally characterizing low biomass samples (Kembel et al., 2012; Oberauner et al., 2012; Mora et al., 2016; Minich et al., 2018b), and thus render relative information alone less useful.

In practice, microbiome samples are often highly variable in terms of total microbial load. Fecal samples are generally characterized by high biomass, while hospital and indoor samples usually present low biomass (Kembel et al., 2012; Mora et al., 2016; McDonald et al., 2018). Low biomass samples impose more challenges to their processing because of contamination and process inefficiencies (Minich et al., 2018b). In fact, in this study we removed *E. coli* sequences from our results since these were frequently detected in our negative controls. We were able to track the corresponding sequences to the DNA polymerase reagent. *E. coli* has been reported as a common molecular biology contaminant from recombinant enzymes such as polymerases (Spangler et al., 2009). We stress that low biomass samples should always be processed with special care, accompanied by negative controls to assess possible contaminations (Salter et al., 2014; Eisenhofer et al., 2018; Goffau et al., 2018).

### Predictive Modeling of Absolute Abundances

Bayesian cumulative probability models (CPM) address the challenges related to absolute abundance estimation naturally by modeling the cumulative probability function of the sample CFU conditional on the observed read counts (Liu et al., 2017). This strategy allows estimation of total microbial load as well as sample absolute abundances of observed bacteria, as it can deal with ordinal outcomes of varying cardinality and even with continuous outcomes. Further, a wealth of outputs information is automatically available upon CPM fitting, e.g., conditional means, quantiles, class and tail probabilities. These quantities can be readily explored to inform decision making with more than simply point estimates, e.g., estimate the probability that a certain critical sample presents with more than a given threshold of CFU for a given taxonomy of interest.

By construction, the model can also generate continuous and (ordinal) categorical predictions (Harrell, 2015). Although the most likely outcome tends to agree with the estimated conditional mean, the former varies as a step function while the latter varies smoothly. The use of Bayesian framework also enables full probabilistic quantification of uncertainty, making interpretation of estimation intervals straightforward. Lastly, by taking advantage of a hierarchical CPM, we show that the method potentially generalizes to previously unseen bacteria, i.e., predicts the sample CFU for taxa that were not primarily included in the modeling step. This is a crucial feature in high-throughput settings as potentially detected taxa are not known a priori. Also, modeling many taxa directly may be financially prohibitive.

Overall, our results indicate that the predictive errors for CFU do not exceed one order of magnitude (on the *log*_*10*_ scale) for observed bacteria. While total microbial load seems more reliably estimated, for both models the absolute errors tend to be no greater than two times the true values–in a reality of logarithmic differences. Whereas one might doubt the importance of estimating 4^∗^10^3^ CFU compared to a true value of 2^∗^10^3^ CFU, our models can still be improved by adding more data points, by considering other predictors related to 16S rRNA amplification, and by including a more diverse set of taxonomies in model fitting.

### Limitations

This work has several limitations toward immediate real-world application. A key modeling limitation is that, even though the hierarchical model has shown relative success at predicting previously unseen bacteria, it has also shown relatively poor performance at predicting sample CFU of *Bacillus cereus* when this taxon was held out during model fitting. This suggests that extrapolation in high-throughput settings may still require modeling more than simply a few taxonomies. Currently, the main laboratorial limitation is the upper limit of quantification caused by the PCR amplification step and its plateau phase. Our method carries the potential to be adapted to address both modeling and laboratorial challenges, which is left for future work.

Another limitation, seemingly inherent to the sample processing method, is the fact that detected sequences might not represent viable bacteria. Still, one can always quantify absolute sample abundance as DNA copies instead of CFU, an alternative to which our method adapts naturally. Additionally, it can be the case that the sampling method (e.g., swab collection) varies widely in area for each sample. In practice, it may be helpful to standardize sample collection according to the target object to be analyzed. Although this should be kept in mind during results interpretation, the impact of such variation is worthy of further investigation. Potentially, the method could be extended to any other organism with any primers other than 16S rRNA V3/V4 region, given that the main achievement of our proposal relies on the equivolumetric library preparation and the employment of samples with varying amounts of DNA that do not saturate the PCR. However, specific primers may vary in terms of amplification efficacy and, hence, in propensity to PCR saturation. This may alter the range of sample CFU or input DNA within which the proportionality between reads and absolute abundance is observed.

Finally, there are practical limitations due to study design, especially related to taxon-specific abundance estimation. First, the employed mock community consists of even proportions of only a few taxa. It is not clear how community complexity may impact the proportionality between observed reads and taxa abundance in general. For instance, taxa that dominate a given sample may disproportionately generate more amplicons, possibly reaching PCR saturation and even affecting less abundant taxonomies. Second, sample collection and surface type may vary according to the application at hand, which cannot be tested using mock communities alone. Whereas it is possible to assess the correlation between estimated total microbial load and the actual microbial load observed in real settings, this is much more difficult to evaluate when it comes to specific taxa.

Future work may involve paired assessment of total microbial load using classical microbiology and HTS techniques in real-world samples, which may render possible to externally validate total microbial load predictions from previously developed models.

## Conclusion

This study has presented an equivolumetric protocol for library preparation prior to 16S rRNA gene amplicon sequencing as well as a modeling strategy to predict sample CFU given HTS observed reads counts. Assuming the same protocol for a set of samples of similar nature, the proposed procedures were shown to recover the proportionality between library sizes and total microbial load–and, more generally, between HTS reads and absolute bacterial abundances within each sample. Still, further research is needed to understand whether such models can generalize to high-throughput settings, in which data from a small subset of taxa are used to make predictions on previously unseen bacteria. Future challenges also involve extending the range of bacterial abundances properly captured by the method and understanding the impact of sample complexity on estimation of taxon-specific abundances. It remains clear, though, that the claims that library size is always an arbitrary sum, often taken for granted by several previous works, and that high-throughput sequencing reads can carry only proportion-based information, are readily overcome by the methods herein proposed.

## Data Availability Statement

All sequence data are deposited in NCBI BioProject PRJNA603167. Data and code to reproduce the analysis are available at https://github.com/biomehub/libsize and as a compressed folder (Supplementary Material 4).

## Author Contributions

GC and LO performed the bioinformatic analysis. GC did the modeling and statistical analysis. AC performed the laboratory experiments and analysis. GC and AC wrote this manuscript. LO performed the revisions. All authors contributed to the article and approved the submitted version.

## Conflict of Interest

All authors are currently full-time employees of BiomeHub (SC, Brazil), a research and consulting company specialized in microbiome technologies. BiomeHub funded the study design, analysis, and data submission for publication.

## Supplementary Material

Supplementary Figure 1 DNA extraction methods are not a limiting factor in the equivolumetric protocol for bacterial absolute abundances recovery in HTS sequencing.

Supplementary Figure 2 Illustration of DNA amplification during PCR reaction for samples of varying input biomass.

Supplementary Table 1 Sequencing information. Metrics and specifications about samples and sequencing run data used in this study.

Supplementary Table 2 R packages.

Supplementary Material 1 Using data from multiple sequencing runs.

Supplementary Material 2 Ordinal Regression predicts total microbial load on CFU scale.

Supplementary Material 3 Ordinal Regression predicts absolute bacterial abundance on CFU scale.

Supplementary Material 4 Compressed folder with data and code.
