# Supplementary material for: Equivolumetric Protocol Generates Library Sizes Proportional to Total Microbial Load in 16S Amplicon Sequencing
Source: Front Microbiol. 2021 Feb 26;12:638231. doi: 10.3389/fmicb.2021.638231 (PMC7952455; doi:10.3389/fmicb.2021.638231)
Supplement: Supplementary Figure 1 — DNA extraction methods are not a limiting factor in the equivolumetric protocol for bacterial absolute abundances recovery in HTS sequencing. [file Data_Sheet_1.PDF]

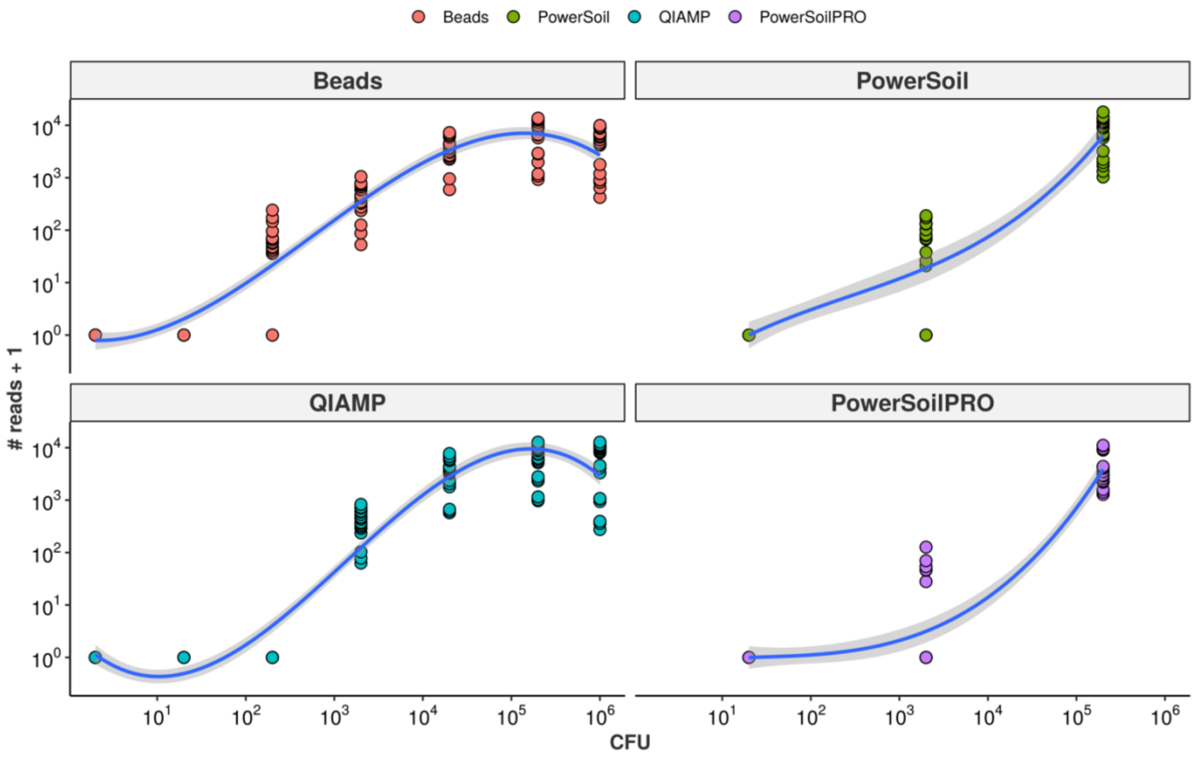

Supplementary figure 1. DNA extraction methods are not a limiting factor in the equivolumetric protocol for bacterial absolute abundances recovery in NGS sequencing. Four different DNA extraction methods were evaluated: **Beads** (Magnetic Beads - Agencourt AMPure XP - purification after thermal lysis) (Beckman Coulter, CA, USA); **QIAMP** QIAamp DNA Mini and Blood Mini; **PowerSoil** DNAeasy Power Soil and **PowerSoilPRO** DNAeasy Power Soil PRO (QIAGEN, Germany). For PowerSoil and PowerSoilPRO not all CFU concentrations were evaluated (8.4 CFU to 8.5x10^6), only three points representing the lowest, medium and highest values were tested. Overall the results indicated that regardless of the DNA extraction method, the NGS sequencing reads recovers the initial microbial load of the samples when using the equivolumetric protocol.

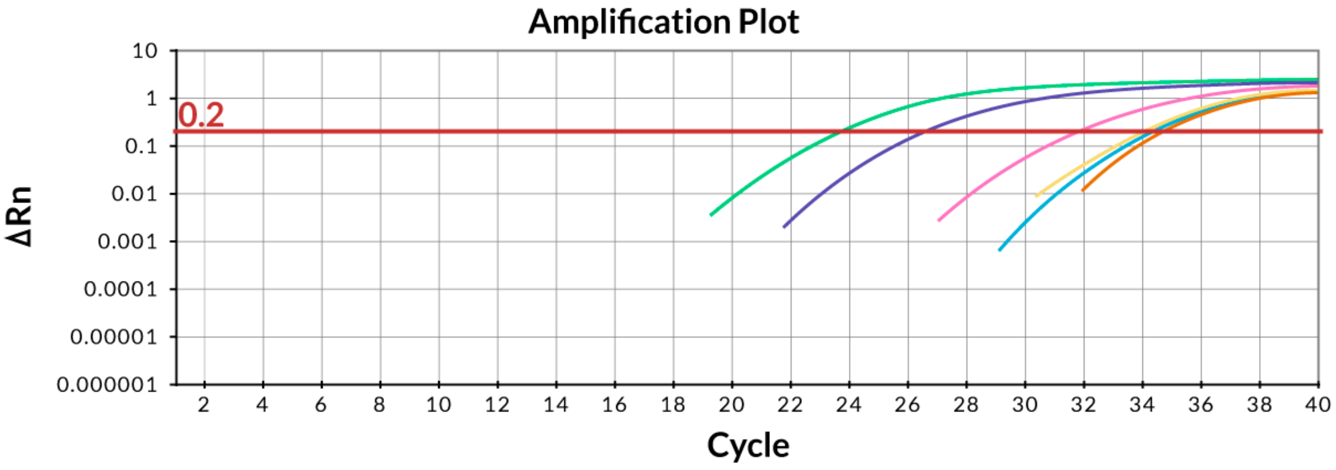

Supplementary figure 2. Illustration of DNA amplification during PCR reaction for samples of varying input biomass. In real-time PCR reactions, we can visualize different input samples reaching a given threshold value (0.2 red line) within different amplification cycles, monitored by increasing fluorescence variation (ΔRN). Sample abundances (CFU) from left to right are represented as: 10^6 – Green curve; 10^5 – Purple curve; 10^4 – Pink curve; 10^3 – Yellow curve; 10^2 – Blue curve and 10^1 – Orange curve. After too many amplification cycles (*e.g.* 38 cycles) we observe that all samples reach the amplification plateau - in which all samples present the same amount of amplified DNA, regardless of input biomass. Using the equivolumetric protocol and reduced number of amplification cycles we can recover the variations in sample inputs as most amplifications are still in the exponential phase of the PCR reaction.

Supplementary table 1. Sequencing run information

Experiment	Seq ID	Date	Kit	Kit Lot	Kit throughput	Reads PF	Clusters PF	Sample Coverage expected	Sample Coverage obtained	PhiX error rate
Synthetic fragment	CF47W	28-Jun-19	V3-600	20339866 20338190	25 M	27,272,423	92.48%	45	53.745	2.29%
HAIMP – Seq1	AR9G6	16-Aug-16	V2-300	20062449 20050998	15 M	*	93.1%	38.045	33.602	*
HAIMP – Seq2	ARC6K	2-Sep-16	V2-300	20062449 20050996	15 M	*	93.1%	29.844	40.531	*
HAIMP – Seq3	ATY5M	24-Feb-17	V2-300	20081917 20086601	15 M	*	*	28.906	29.228	*
HAIMP – Seq4	AY0WK	26-May-17	V2-300	20106090 20116131	15 M	*	92.4%	26.875	28.777	*
HAIMP – Seq5	B47J9	20-Jun-17	V2-300	20133531 20140742	15 M	*	94.90%	26.172	21.817	*
HAIMP – Seq6	AP4Y6	16-Sep-16	V2-300	20053902 20047016	15 M	*	90.80%	25.5	17.657	*
HAIMP – Seq7	AR9J7	30-Aug-16	V2-300	20062449 20050996	15 M	*	93.8%	17.89	15.459	*
HAIMP – Seq8	G19PG	20-Jun-17	V2-300 Micro	20161114 20123715	4 M	*	94.4%	16.615	16.856	*
HAIMP – Seq9	ARLG5	27-Jan-17	V2-300	20067980 20076225	15 M	*	92.0%	16.485	19.428	*
HAIMP – Seq10	AP6JV	9-Sep-16	V2-300	20053896 20050996	15 M	*	94.3%	16.175	14.405	*
HAIMP – Seq11	B449L	1-Aug-17	V2-300	20133531 20140742	15 M	*	94.1%	11.745	8.475	*
HAIMP – Seq12	G158L	22-Feb-17	V2-300 Micro	20088731 20126864	4 M	*	94.4%	11.38	12.416	*
HAIMP – Seq13	AYJPM	12-Jun-17	V2-300	20123715 20115806	15 M	*	94.4%	7.891	8.886	*
HAIMP – Seq14	ADC84	30-Apr-15	V2-300	*	15 M	*	*	60	61.744	*
ATCC – Seq1	CGB23	26-Jul-19	V3-600	20345776 20351090	25 M	24,440,428	90.95%	45	43.527	2.61%
ATCC – Seq2	CHW9T	16-Aug-19	V3-600	20357983 20345731	25 M	23,985,332	92.14%	29.7	33.123	2.67%
ATCC – Seq3	CJVYY	23-Aug-19	V3-600	20365653 20360395	25 M	26,025,704	88.90%	28.15	29.184	2.69%
ATCC – Seq4	CJ3LD	27-Sep-19	V2-300	20357979 20372379	15 M	13,155,728	93.26%	15	15.335	2.68%
ATCC - Extractions	CKHGJ	28-Aug-19	V3-600	20369353 20367211	25 M	25,419,260	90.76%	45	44.078	2.96%

* missing information

Obs. before 2018 we didn't use PhiX in all the sequencing runs.

Experimental data record improved all over the years, however the results maintained the good yield from 2015 to 2019.

Minimal variations could be caused by the pool quantification and fragment size adjustment.

Error rates and Clusters PF are highly dependent on the sequencing context, giving the majority of sequencing pools are from amplicons, which may lower the sequencing diversity.

Standard sequencing sample coverage for built environments is 45,000, however in these experiments coverages were lowered on purpose to test for diversity an sequencing recovery, as well as the normalization process.

Regardless the sequencing kit used, all runs were performed as single-end 300pb.

Supplementary table 2. References from software packages

R Package	Reference
brms	Bürkner, P.-C. & Others. brms: An R package for Bayesian multilevel models using Stan. J. Stat. Softw. 80, 1–28 (2017).
caret	Kuhn, M. caret: Classification and Regression Training. (2020).
DescTools	Signorell, A. DescTools: Tools for Descriptive Statistics. (2020).
docstring	Kurkiewicz, D. docstring: Provides Docstring Capabilities to R Functions. (2017).
furrr	Vaughan, D. & Dancho, M. furrr: Apply Mapping Functions in Parallel using Futures. (2018).
future	Bengtsson, H. future: Unified Parallel and Distributed Processing in R for Everyone. (2020).
ggpubr	Kassambara, A. ggpubr: 'ggplot2' Based Publication Ready Plots. (2019).
ggrepel	Slowikowski, K. ggrepel: Automatically Position Non-Overlapping Text Labels with 'ggplot2'. (2019).
ggridges	Wilke, C. O. ggridges: Ridgeline Plots in 'ggplot2'. (2020).
knitr	Xie, Y. knitr: A general-purpose Tool for dynamic report generation in R. R package version 1, (2013).
latex2exp	Meschiari, S. latex2exp: Use LaTeX Expressions in Plots. (2015).
modelr	Wickham, H. modelr: Modelling Functions that Work with the Pipe. (2019).
patchwork	Pedersen, T. L. patchwork: The Composer of Plots. (2019).
phyloseq	McMurdie, P. J. & Holmes, S. phyloseq: An R package for reproducible interactive analysis and graphics of microbiome census data. PLoS ONE vol. 8 e61217 (2013).
plotly	Sievert, C. plotly for R. (2018).
plyr	Wickham, H. The Split-Apply-Combine Strategy for Data Analysis. Journal of Statistical Software vol. 40 1–29 (2011).
rafalib	Irizarry, R. A. & Love, M. I. rafalib: Convenience Functions for Routine Data Exploration. (2015).
RColorBrewer	Neuwirth, E. RColorBrewer: ColorBrewer Palettes. (2014).
Rcpp	Eddelbuettel, D. et al. Rcpp: Seamless R and C++ integration. J. Stat. Softw. 40, 1–18 (2011).
readr	Wickham, H., Hester, J. & Francois, R. readr: Read Rectangular Text Data. (2018).
rlang	Henry, L. & Wickham, H. rlang: Functions for Base Types and Core R and 'Tidyverse' Features. (2020).
rms	Harrell, F. E., Jr. rms: Regression Modeling Strategies. (2019).
scales	Wickham, H. & Seidel, D. scales: Scale Functions for Visualization. (2019).
splines	R Core Team. R: A Language and Environment for Statistical Computing. (2019).
tidybayes	Kay, M. tidybayes: Tidy Data and Geoms for Bayesian Models. (2020) doi:10.5281/zenodo.1308151.
tidyverse	Wickham, H. et al. Welcome to the tidyverse. Journal of Open Source Software vol. 4 1686 (2019).
