# Supplementary material for: Equivolumetric Protocol Generates Library Sizes Proportional to Total Microbial Load in 16S Amplicon Sequencing
Source: Front Microbiol. 2021 Feb 26;12:638231. doi: 10.3389/fmicb.2021.638231 (PMC7952455; doi:10.3389/fmicb.2021.638231)
Supplement: Supplementary Material 4 — Compressed folder with data and code. [file Data_Sheet_5.ZIP › Supplementary_Material_4/README.pdf]

Equivolumetric protocol generates library sizes proportional to total microbial load in 16S amplicon sequencing

Repository with data and code for the analyses involved in the corresponding manuscript (https://doi.org/10.1101/2020.02.03.932301).

- All Supplementary Materials .Rmd files use `data/normalized/phyloseq.rds` as primary input.
- Within each .Rmd file, you will need to set the variable `REFIT = TRUE` in order to fit the models and save corresponding (quite big) .rds files.
- Once the .rds files for the fitted models and cross-validations exist, you can just set `REFIT = FALSE` and `knit` the .Rmd files to produce beautiful .html reports.

All sequence data are deposited in NCBI BioProject PRJNA603167.

Dependencies:

R v. 3.6.3

brms v. 2.14.4

docstring v. 1.0.0

furrr v. 0.2.2

ggpubr v. 0.4.0

ggridges v. 0.5.3

latex2exp v. 0.4.0

modelr v. 0.1.8

patchwork v. 1.1.1

phyloseq v. 1.30.0

RColorBrewer v. 1.1.2

Rcpp v. 1.0.6

rlang v. 0.4.10

rms v. 6.1.0

tidybayes v. 2.3.1

tidyverse v. 1.3.0

caret v. 6.0.86

future v. 1.21.0

DescTools v. 0.99.39

rafalib v. 1.0.0

scales v. 1.1.1

plyr v. 1.8.6

ggrepel v. 0.9.1

knitr v. 1.31
